# Three-Dimensional High-Resolution Magnetic Resonance Imaging for the Assessment of Cervical Artery Dissection

**DOI:** 10.3389/fnagi.2022.785661

**Published:** 2022-07-05

**Authors:** Xianjin Zhu, Yi Shan, Runcai Guo, Tao Zheng, Xuebin Zhang, Zunjing Liu, Kunpeng Liu

**Affiliations:** ^1^Department of Neurology, Beijing Friendship Hospital, Capital Medical University, Beijing, China; ^2^Graduate School of Peking Union Medical College, Beijing, China; ^3^Department of Neurology, China-Japan Friendship Hospital, Beijing, China; ^4^Department of Radiology, China-Japan Friendship Hospital, Beijing, China; ^5^Department of Anesthesiology, Peking University International Hospital, Beijing, China

**Keywords:** cervical artery dissection, intimal flap, high resolution magnetic resonance image, intramural hematoma, double lumen

## Abstract

**Background and Purpose:**

Diagnosing cervical artery dissection (CAD) is still a challenge based on the current radiographic criteria. This study aimed to assess the value of three-dimensional high-resolution magnetic resonance imaging (3D HRMRI) in the detection of the signs of CAD and its diagnosis.

**Materials and Methods:**

Patients with CAD from January 2016 to January 2021 were recruited from our 3D HRMRI database. The signs of dissection (intramural hematomas, intimal flap, double lumen), length and location of the dissection, thickness of the intramural hematoma, intraluminal thrombus, and percentage of dilation of the outer contour of the dissection on 3D HRMRI were assessed.

**Results:**

Fourteen patients with 16 CADs, including 12 carotid CADs and 4 vertebral CADs, were finally diagnosed in this study. On 3D HRMRI, intramural hematomas were detected in 13/16 (81.3%) lesions with high sensitivity (100%) and high specificity (100%). Intimal flaps were found in 9/16 (56.3%) lesions with moderate sensitivity (64.3%) and high specificity (88.9%). Double lumen signs were observed in 4/16 (25.0%) lesions with high sensitivity (80.0%) and high specificity (100%). In addition, concomitant intraluminal thrombus were detected in 4/16 (25.0%) lesions with high sensitivity (80.0%) and high specificity (100%). The mean length of dissection was (25.1 ± 13.7) mm. The mean thickness of the intramural hematoma was (4.3 ± 2.3) mm. The mean percentage of dilation for the outer contour of the dissection was (151.3 ± 28.6)%.

**Conclusion:**

The 3D HRMRI enables detection of the dissecting signs, such as intramural hematoma, intimal flap, double lumen, and intraluminal thrombus with high sensitivity and specificity, suggesting a useful, and non-invasive tool for definitively diagnosing CAD.

## Introduction

Cervical arterial dissection (CAD), involving the extracranial carotid or vertebral artery, is an important cause of stroke (Debette and Leys, 2009) with the major mechanism being artery-to-artery embolism (Morel et al., 2012) associated with intimal damage and microthrombosis (Wu et al., 2019). Optimum treatment of CAD strongly relies on the accuracy of the diagnosis (Markus et al., 2019). The difficulties in the diagnosis of CAD have been well-documented in many previous studies (Robertson and Koyfman, 2017; Markus et al., 2019). Its clinical symptoms are often non-specific (Gallerini et al., 2019). Digital subtraction angiography (DSA), which was regarded as the gold standard for luminal imaging before magnetic resonance imaging (MRI), is no longer recommended for the diagnosis of CAD, not only because of its invasive nature but also because it lacks the ability to visualize the vessel wall (Debette and Leys, 2009). Computed tomography angiography (CTA) and MR angiography (MRA) are non-invasive techniques for assessing CAD (Caplan, 2008; Provenzale, 2009) but these modalities also focus on the luminal characteristics without providing enough information on the vessel wall (Debette and Leys, 2009). Doppler sonography is another non-invasive technique for assessing carotid atherosclerotic plaque (Lyu et al., 2020; Goudot et al., 2021) and for diagnosing CAD sometimes (Caplan, 2008; Robertson and Koyfman, 2017) but it should only be considered as a screening tool due to its operator dependence and poor diagnostic yield (Debette and Leys, 2009). Understanding the pathognomonic vessel wall radiological features of CAD, such as a double lumen, intimal flap, and intramural hematoma, is of utmost importance in making the definitive diagnosis (Debette and Leys, 2009).

Currently, MRI has become the first-line diagnostic modality for CAD (Debette and Leys, 2009). High-resolution MRI (HRMRI) has been used in the previous studies because it offers good visualization of the arterial wall (Yin, 2017; Zhao and Zhu, 2021) making it possible to detect the features of arterial dissection with greater sensitivity (Eliasziw et al., 1994; Bachmann et al., 2007; Saam et al., 2009; Qiao et al., 2011, 2016; Hunter et al., 2012; Machet et al., 2013; Yamada et al., 2016; Zhu et al., 2016, 2020; Coppenrath et al., 2017; McNally et al., 2018; Huang et al., 2019). This technique often involves black blood two-dimensional (2D) fast spin echo (FSE) sequences that rely mostly on double inversion recovery preparation or pre-regional saturation pulses to null the blood signal (Saam et al., 2009; Hunter et al., 2012; Machet et al., 2013; Coppenrath et al., 2017; Yin, 2017). The limitations of this technique include having a limited number of slices due to time constraints and anisotropic spatial resolution with low spatial resolution in the slice-select direction (Qiao et al., 2011; Zhu et al., 2016; Yin, 2017). It could be challenging to radiologically diagnose CAD using 2D HRMRI because of the features of the cervical carotid or vertebral artery, such as the variability of the dissecting length and the characteristics of the signals on MRI, the tortuous course of the lesions, adjacent veins, the smaller caliber of the vertebral artery, and the presence of an intraluminal thrombus (Debette and Leys, 2009; Morel et al., 2012).

Recently, three-dimensional (3D) HRMRI with variable flip-angle refocusing pulse sequence was introduced. In contrast to 2D HRMRI, 3D HRMRI has many advantages such as a higher signal–to-noise ratio, a larger coverage, and reduced scanning time (Qiao et al., 2011). Furthermore, 3D HRMRI could acquire isotropic volumetric datasets with sub-millimeter voxel size, which could enable multiplanar reconstruction for a more global assessing of vessel wall (Zhu et al., 2020). This 3D HRMRI technique has been used to image carotid and intracranial arterial walls for the evaluation of atherosclerotic plaque and intracranial dissecting aneurysms (Qiao et al., 2016; Zhu et al., 2016, 2020) and it may be also suitable for detecting the signs of dissection in the cervical carotid or vertebral artery.

## Materials and Methods

### Patient Selection

We reviewed our HRMRI database for patients with CAD diagnosed using imaging techniques from January 2016 to January 2021. Considering no widely recognized golden standard available for the diagnosis of CAD nowadays, a definitive diagnosis of CAD was made when any pathognomonic sign of dissection (double lumen, intimal flap, and intramural hematoma) was detected at least one imaging modality such as MRA, CTA, DSA, or 3D HRMRI. Diffusion-weighted imaging (DWI) and HRMRI were performed simultaneously. This observational cross-sectional study was approved by our local institutional ethics committee. The demographics, clinical findings, imaging data, and risk factors—such as hypertension, hyperlipidemia, diabetes mellitus, and cigarette smoking—were noted.

### Imaging Protocol

All MR examinations were performed with a 3T MRI scanner (Ingenia; Philips Healthcare, Nederland) using a 20-channel integrated head/neck coil. 3D HRMRI was acquired using a pre- and post-contrast black blood technique called volumetric isotropic turbo spin-echo acquisition (VISTA). The parameters were as follows: repetition time/echo time = 800/21 ms, field of view = 180 × 180, matrix = 300 × 300, NEX = 1, and slice thickness = 0.6 mm; the voxel volume was 0.6 mm × 0.6 mm × 0.6 mm on the T1-weighted image (T1WI). The post-contrast T1WI was obtained 5 min after gadolinium injection (0.1 mmol/kg of gadopentetate dimeglumine, Magnevist, Berlin, Germany) with the same parameters as pre-contrast T1WI.

### High-Resolution Magnetic Resonance Imaging Assessment

Initially, two trained readers with more than 5 years of experience in reading HRMR images and blinded to the clinical information and DWI of the patients assessed for the signs of dissection, such as intramural hematoma, intimal flap, double lumen, and intraluminal thrombus on the VISTA images. Analytical data were used to calculate the interobserver variability. The differences between the two observers were solved by consensus.

**FIGURE 1:**
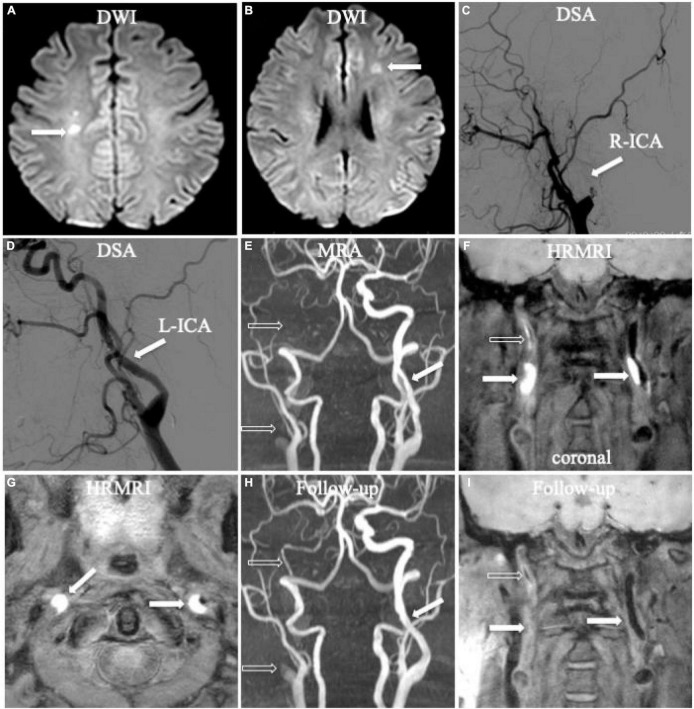
Bilateral acute ischemic stroke associated with dissection in the internal carotid arteries (ICA) bilaterally. Diffusion-weighted imaging (DWI) showed acute ischemic stroke in bilateral hemispheres (**A,B**, arrow). Digital subtraction angiography (DSA) only showed occlusion in the right ICA (**C**, arrow) and mild irregular stenosis in the left ICA (**D**, arrow) without any direct dissecting signs. Initial magnetic resonance angiography (MRA) displayed occlusion (**E**, empty arrow) in the right ICA and mild irregular stenosis in the left ICA (**E**, arrow), similar as the founding on DSA. Initial coronal T1-weighted high-resolution magnetic resonance imaging (HRMRI) demonstrated a T1-hyperintense intramural hematoma (**F**, arrow) in the proximal C1 segments of the ICA bilaterally and a T1-hyperintense intraluminal thrombus in the middle and distal right ICA (**F**, empty arrow). Transverse HRMRI presented with typical crescent-shaped intramural hematomas (**G**, arrow). Follow-up MRA showed partial recanalization in the middle and distal right ICA (**H**, empty arrow) and a nearly normal lumen in the left ICA (**H**, arrow). Follow-up coronal HRMRI exhibited disappearance of the T1-hyperintense intramural hematoma (**I**, arrow) in the ICA bilaterally and T1-hyperintense intraluminal thrombus (**I**, empty arrow) in the right ICA.

**FIGURE 2:**
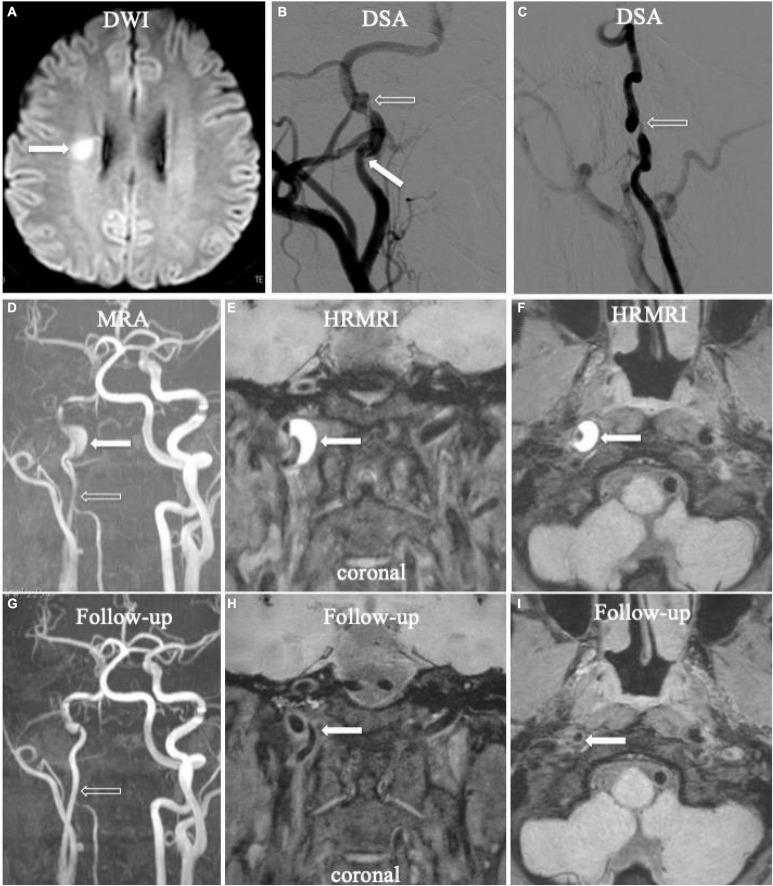
Acute ischemic stroke (**A**, arrow) in the territory of the right ICA. Digital subtraction angiography (DSA) showed aneurysmal dilation with focal stenosis (**B,C**, empty arrow) and suspicious intimal flap (**B**, arrow) in the right ICA. Initial MRA displayed tapered stenosis (**D**, empty arrow) and a suspicious hematoma (**D**, arrow). Initial HRMRI showed an intramural hematoma on the T1-weighted coronal (**E**, arrow) and transverse image (**F**, arrow). Follow-up MRA (**G**, arrow) showed partial recanalization on the affected arterial lumen. The intramural hematoma had disappeared on follow-up T1-weighted coronal (**H**, arrow) and transverse (**I**, arrow) HRMRI.

**FIGURE 3:**
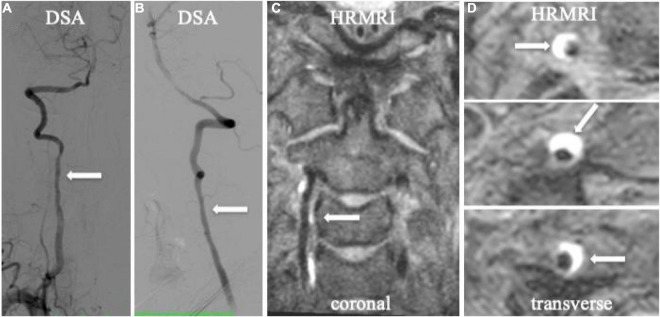
Spiral intramural hematoma in the patient with vertebral artery dissection. Digital subtraction angiography (DSA) showed irregularly mild stenosis without any dissecting signs on anteroposterior and lateral projections (**A,B**, arrow). T1-weighted HRMRI showed an spiral intramural hematoma in the right vertebral artery in the coronal (**C**, arrow) and transverse planes (**D**, arrow).

Intramural hematoma was defined as the detection of crescent-shaped, intermediate-to-high signal intensity within the arterial wall on pre-contrast T1WI (Figures 1–3; Wu et al., 2019; Zhu et al., 2020). Furthermore, signal intensity alterations were classified as homogenous or heterogeneous (Wu et al., 2019; Zhu et al., 2020). Intraluminal thrombus was defined as hyper-intense filling on pre-contrast T1W images (Wu et al., 2019) and an intimal flap was defined as an abnormal curvilinear or linear structure separating a true lumen from a false lumen (Zhu et al., 2020). To distinguish the intimal flap from the inflow artifact that usually appears at the center portion of the lumen, we considered a linear structure as an intimal flap that extended to the arterial sidewall on pre- and/or post-contrast T1W images (Wu et al., 2019; Zhu et al., 2020). The double-lumen sign was defined as two jets of flow void within one vessel on MRI (Wu et al., 2019; Zhu et al., 2020).

The stenosis degree at the most stenotic site of the true lumen was defined as (1-lesion luminal diameter/reference luminal diameter) × 100%, according to the North American Symptomatic Carotid Endarterectomy Trial criteria (Eliasziw et al., 1994). The degree of stenosis was graded as no or mild stenosis (<50%), moderate stenosis (50–69%), and severe stenosis or occlusion (70–100%). Enlargement of the outer contour of dissection was assessed using the following formula: percentage of dilation = *D*_*dilatation*_/*D*_*normal*_ × 100 (Itabashi et al., 2014). The *D*_*dilatation*_ was defined as the maximum diameter of the outer contour of the lesions and *D*_*normal*_ as the outer diameter of the nearest normal segment proximal to the lesions. If the proximal segment was not available, the outer diameter of the nearest normal segment distal to the lesions was substituted. To assess interobserver variability, the two readers independently measured the *D*_*dilatation*_ and *D*_*normal*_ of all the lesions.

### Statistical Analysis

Continuous variables were summarized as mean ± standard deviation (*SD*). Categorical variables were presented as percentages. The Cohen’s *k* coefficient was computed to quantify the interobserver agreement for detecting intramural hematoma, intimal flap, double lumen, intraluminal thrombus, and the heterogeneous signal intensity of the intramural hematoma. A value of *k* > 0.75 was used to indicate a high level of agreement, and 0.40 ≤ *k* ≤ 0.75 denoted moderate agreement. Intraclass correlation coefficient (ICC) was used to determine interobserver agreement for the measurements of the length of dissection, thickness of intramural hematoma, and percentage of dilation. The SPSS 20 (SPSS, Inc., Chicago, IL) was used for the statistical analysis. All reported *p*-values were 2-sided, and *p*-values < 0.05 were considered significant.

## Results

### Patients

A total of 14 patients with 16 CADs (6 men and 8 women) were enrolled in this study, including 7 patients who presented with acute ischemic stroke and 7 patients without acute ischemic stroke but with neurological symptoms (Table 1). The mean age was 43.9 ± 10.9 years. Among the 14 patients, 9 patients presented with unilateral internal carotid arteries (ICA) dissections (64.3%), 3 with unilateral vertebral dissections (21.4%), 2 with multiple dissections (14.3%)—including 1 patient with bilateral ICA dissection and 1 with unilateral ICA and vertebral dissection. Of the 16 lesions [12 lesions (75.0%) in the extracranial carotid artery, 4 lesions (25.0%) in the extracranial vertebral artery] (Figure 2), dissections caused no or mild stenosis in 6 lesions, moderate stenosis in 4 lesions, and severe stenosis or occlusion in 6 lesions. The risk factors included hypertension (*n* = 2, 14.3%), hyperlipidemia (*n* = 1, 7.1%), diabetes mellitus (*n* = 1, 7.1%), smoking (*n* = 5, 35.7%), and a history of trauma to the neck within the previous 28 days (*n* = 2, 14.3%).

**TABLE 1:** Clinical characteristics of the patients with cervical artery dissection.

Patient no.	Symptoms	Risk factors	NIHSS	mRS
1	Headache	-	0	0
2	Recurrent dizziness	Hyperlipidemia	0	0
3	Left lower extremity weakness and numbness	Smoking	2	1
4	Left upper extremity weakness and numbness	-	3	1
5	Left upper extremity weakness and slurring of speech	Smoking	3	1
6	Recurrent dizziness	Smoking	0	0
7	Recurrent dizziness and headache	-	0	0
8	Right extremity weakness and slurring of speech	-	3	1
9	Slurring of speech and facial palsy	History of trauma	3	1
10	Right hand weakness and slurring of speech	History of trauma	3	1
11	Dizziness	-	0	0
12	Right side weakness	-	1	1
13	Left side weakness and slurring of speech	Hypertension, smoking	3	1
14	Recurrent dizziness	Hypertension, diabetes mellitus, smoking	1	1

*NIHSS, National Institutes of Health Stroke Scale; mRS, modified Rankin Scale.*

### Interobserver Agreement

The interobserver agreement was high for the identification of intramural hematomas (*k* = 0.818), intimal flaps (*k* = 0.871), and the double lumen sign (*k* = 0.846); it was moderate for identifying intraluminal thrombi (*k* = 0.714); and it was high for the measurements of the length of dissection (*ICC* = 0.919), thickness of intramural hematoma (*ICC* = 0.994), and percentage of dilation (*ICC* = 0.919).

### High-Resolution Magnetic Resonance Imaging Assessment

**FIGURE 4:**
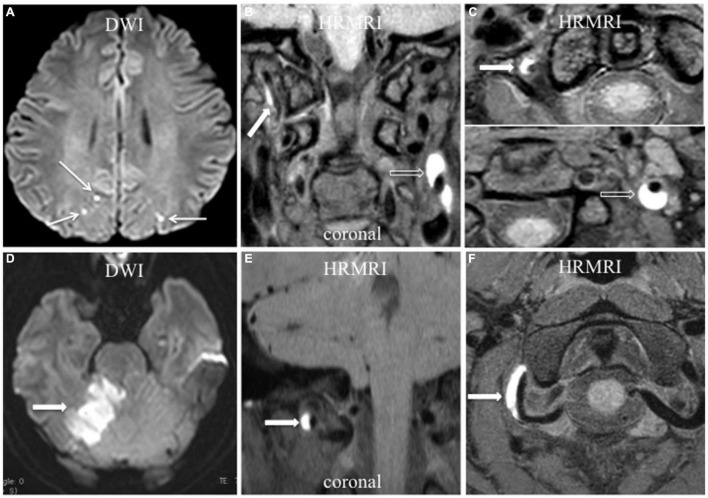
Acute ischemic stroke associated with vertebral artery dissection. **(A–C)** Multiple acute ischemic stroke lesions in the parietooccipital junction bilaterally (**A**, arrow) with dissection in the right vertebral artery and left ICA. T1-weighted HRMRI showed an intramural hematoma in the right vertebral artery (**B,C**, arrow) and left ICA (**B,C**, empty arrow) in the coronal and transverse planes. **(D–F)** Acute ischemic stroke in the right cerebellum (**D**, arrow) with V3 segment dissection of the right vertebral artery. T1-weighted HRMRI showed an intramural hematoma in the right vertebral artery in the coronal (**E**, arrow), and transverse (**F**, arrow) planes.

On 3D HRMRI, intramural hematomas were identified in 13 lesions (81.3%) in 11 patients (Figures 1–4) with the sensitivity 100% and the specificity 100%. Among the 11 patients, 6 presented with unilateral ICA intramural hematomas, 3 with unilateral vertebral intramural hematomas, 1 with bilateral ICA intramural hematomas, and 1 with unilateral ICA and vertebral intramural hematomas. Of the 13 lesions with intramural hematomas, 7 (53.8%) showed heterogeneous signal intensity. The mean thickness of the intramural hematomas was (4.3 ± 2.3) mm. Intraluminal thrombi were found in four ICA dissections (25.0%) of four patients (sensitivity 80.0%, specificity 100%) (Figures 1–4).

On 3D HRMRI, the intimal flap was detected in nine lesions (56.3%) in nine patients (sensitivity 64.3%, specificity 88.9%), including eight intimal flaps in unilateral ICA of eight patients and an intimal flap in unilateral vertebral dissection of one patient. A double lumen was detected in four lesions (25.0%) in four patients (sensitivity 80.0%, specificity 100%), including three unilateral ICA dissections in three patients and one vertebral dissection in one patient (Table 2).

**TABLE 2:** Signs of dissection on high-resolution magnetic resonance imaging (HRMRI) for individual patients and lesions.

Patient no.	Lesion location	Initial HRMRI			Follow-up HRMRI	
		Intramural hematoma	Intimal flap	Double lumen	Lumen	Intramural hematoma
1	LICA	Y	Y	N	-	-
	RVA	Y	N	N	-	-
2	RICA	Y	N	N	-	-
3	RICA	Y	N	N	Partial recanalization	Reduced
	LICA	Y	N	N	Complete recanalization	Disappeared
4	RICA	Y	Y	N	-	-
5	RICA	Y	Y	Y	Partial recanalization	Disappeared
6	RVA	Y	N	N	Complete recanalization	Disappeared
7	RVA	Y	N	N	-	-
8	LICA	N	Y	Y	-	-
9	LICA	Y	Y	N	Complete recanalization	Disappeared
10	LICA	Y	Y	N	Complete recanalization	Disappeared
11	RICA	Y	N	N	Complete recanalization	Disappeared
12	RVA	Y	Y	Y	-	-
13	LICA	N	Y	Y	-	-
14	RICA	N	Y	N	-	-

*LICA, left internal carotid artery; RICA, right internal carotid artery; RVA, right vertebral artery; Y, yes; N, no; HRMRI, high-resolution magnetic resonance imaging.*

In total, three signs of dissection were detected in 2 lesions, two signs in 7 lesions, and one sign in 7 lesions on 3D HRMRI. The mean length of dissection was (25.1 ± 13.7) mm. The mean percentage of dilation for the outer contour of the dissection was (151.3 ± 28.6%).

Follow-up 3D HRMRI was performed in six patients with seven lesions (mean 204.8 ± 109.9 days) (Figures 1, 2). On follow-up 3D HRMRI, intramural hematoma disappearance was found in 6/7 lesions (85.7%), including luminal complete recanalization in 5/7 lesions (71.4%) and luminal partial recanalization in 1/7 lesions (14.3%). For one lesion (14.3%), the intramural hematoma was reduced and became an intermediate signal intensity with luminal partial recanalization (Table 2).

## Discussion

This study showed that 3D HRMRI with pre- and post-contrast T1-weighted images was useful for investigating the pathognomonic radiological features of cervical dissection, such as intramural hematoma, intimal flap, double lumen, and intraluminal thrombus with high sensitivity and specificity, thus allowing the definitive diagnosis of dissection.

Intramural hematoma was the most specific sign of CAD (carotid or vertebral), with the typical imaging manifestation of crescent-shaped intermediate-to-high signal intensity surrounding a lumen within the arterial wall on a pre-contrast T1-weighted image. In this study, intramural hematoma was the most frequent sign of dissection detected on 3D HRMRI, with a positive rate of 81.3%. Intramural hematomas were best revealed in the subacute or early chronic stage with high signal intensity; however, after 2 months, most of them became isointense and became difficult to recognize on MR images (Habs et al., 2011). The disappearance of intramural hematomas with regression of dissection has been found in over 80% of cases at radiological follow-up, and the mean time for the arterial lumen to return to normal is approximately 3 months (Morel et al., 2012). In this study, intramural hematomas disappeared in 6/7 lesions (85.7%) at 3D HRMRI follow-up with complete or partial recanalization.

Intraluminal thrombi might play a major role in the pathogenesis of stroke, with the radiological characteristics of an intraluminal filling defect within the lumen on MRA (Yamada et al., 2016) or a hyper-intense filling defect within the lumen on pre-contrast T1W images (McNally et al., 2018). An intraluminal thrombus can be detected on the dissecting site (Markus et al., 2019) or the distal segment of the dissecting vessel (McNally et al., 2018). The 3D HRMRI could provide large coverage and a high signal-to-noise ratio and improve the ability to assess intraluminal thrombi in dissecting vessels.

An intimal flap, with or without a double lumen, was also regarded as a reliable diagnostic finding for CAD (Debette and Leys, 2009). However, it is a subtle sign, and it is only seen in a minority of cases. We found that the intimal flap could be well identified on the post-contrast HRMR T1-weighted images—it manifested as an enhanced curvilinear or linear structure (Debette and Leys, 2009). Detection of the double-lumen sign relied on the identification of blood products in the false lumen (Morel et al., 2012). An entry-exit-type dissection with a communication between the true and false lumen would provide a constant flow of blood through the false lumen, resulting in a signal flow-void similar to that of the true lumen on MRI (Mizutani et al., 2001). In contrast, another type of dissection without a communication between the true and false lumen often has an intramural hematoma in the false lumen, with various signal intensities according to the hemorrhagic stage on MRI (Habs et al., 2011; Zhu et al., 2020).

Vertebral CADs were less commonly reported than carotid CADs in previous studies (Babo von et al., 2013; Traenka et al., 2017). One of the main reasons for the accurate diagnosis of vertebral CAD is the small size of the vertebral arteries and the influence of bony structures (Debette and Leys, 2009). With the increased use of MRI, vertebral CAD is more frequently reported. The 3D HRMRI could be useful in cases of suspected vertebral CAD or multiple lesions (Figure 3).

High-resolution magnetic resonance imaging has been used to reveal the arterial structure for detecting the signs of dissection, mainly with 2D acquisition (Machet et al., 2013; Yin, 2017). However, the reliable identification of arterial dissection by 2D images might be impaired by the lower spatial resolution in the slice-select direction and the limited anatomic coverage (Yin, 2017). Multiple CADs were found in 13–16% of cases, which emphasized the significance of broad coverage imaging. Compared with 2D imaging, 3D HRMRI enables the coverage of a larger volume of cervical arteries and can provide isotropic reconstructed images (Bachmann et al., 2007; Yamada et al., 2016; McNally et al., 2018; Huang et al., 2019). It has the advantage of evaluating the complex anatomy of artery dissections, especially in cases with multiple lesions.

Our study had several limitations. First, it only included a small number of subjects, which may limit the generalizability of the results. Second, not all imaging follow-up findings were available. Finally, the diagnosis was not confirmed by histological examination. Further studies with more cases and follow-up information are needed to assess the sensitivity and specificity of 3D HRMRI, and reveal the imaging changes in 3D HRMRI findings.

## Conclusion

The 3D HRMRI enables a larger anatomical coverage with high isotropic spatial resolution and high reproducibility to reliably assess the signs of dissection—such as intramural hematoma, intimal flap, double lumen, and a concomitant intraluminal thrombus with high sensitivity and specificity—suggesting a useful and non-invasive tool for definitively diagnosing CAD.

## Data Availability Statement

The original contributions presented in this study are included in the article/supplementary material, further inquiries can be directed to the corresponding authors.

## Ethics Statement

The studies involving human participants were reviewed and approved by the Ethics Committee of clinical trials of drugs or devices in China-Japan Friendship Hospital. The patients/participants provided their written informed consent to participate in this study. Written informed consent was obtained from the individual(s) for the publication of any potentially identifiable images or data included in this article.

## Author Contributions

XJZ: manuscript writing, analysis, interpretation of results, and editing. YS: manuscript writing, analysis, data acquisition, and participant recruitment. ZL and KL: research design, interpretation of results, and manuscript writing and editing. RG, TZ, and XBZ: data acquisition, participant recruitment, image processing, and editing. All authors contributed to the manuscript and approved the submitted version.

## Conflict of Interest

The authors declare that the research was conducted in the absence of any commercial or financial relationships that could be construed as a potential conflict of interest. The reviewer QY declared a shared parent affiliation with the authors, XJZ to the handling editor at the time of review.

## Publisher’s Note

All claims expressed in this article are solely those of the authors and do not necessarily represent those of their affiliated organizations, or those of the publisher, the editors and the reviewers. Any product that may be evaluated in this article, or claim that may be made by its manufacturer, is not guaranteed or endorsed by the publisher.
